# The Post-Pandemic World: between Constitutionalized and Authoritarian Orders – China’s Narrative-Power Play in the Pandemic Era

**DOI:** 10.1007/s11366-020-09695-3

**Published:** 2020-10-12

**Authors:** Yung-Yung Chang

**Affiliations:** International Consortium for Research in the Humanities, Friedrich-Alexander-Universität Erlangen-Nürnberg (FAU), Hartmannstr. 14, 91052 Erlangen, Germany

**Keywords:** Narrative power, Strategic narratives, Soft power, Foreign diplomacy, China, COVID-19 pandemic, (Dis)information, World order, Authoritarianism

## Abstract

The paper aims to address the development of China’s narrative power during the COVID-19 pandemic and its impact on world order. It argues that in the post-pandemic world, the emergence of the authoritarian sub-order would be prompted by China’s more proactive narrative power, given that the climate of opinion is ambiguous when faced with the uncertainty of the pandemic. (This does not imply the end of the existing liberal order; instead, it features the coexistence of both orders.) To understand how China’s narrative power has encouraged the emergence of the authoritarian sub-order to coexist in parallel with the dominant constitutional order, the article first reviews the existing literature concerning the changing world order. In this section, it also briefly defines and differentiates between the constitutional and authoritarian orders, what defines world order, and what distinguishes authoritarian from constitutional liberal order. Second it looks at the theoretical grounding. The nature, role and power of narratives are explored. Ideas about strategic narratives and the economics of attention are discussed. This theoretical background paves the way to examine China’s narrative power during a pandemic. Lastly, it switches to the Chinese perspective to address its support for the plurality of orders and its awareness of the strength of narrative in influencing dominant ideas. It looks at how China’s narrative power has been exercised from three perspectives (formation, projection and reception). Here, it mainly tackles how China has used its narrative power to spin the pandemic to its advantage in the reorganization of world order: improving its international image and advocating the authoritarian order as an alternative. China has been building its narrative along with its changing strategic diplomacy – from restrained and low-profile to proactive and assertive. In the conclusion, some reflections on China’s narrative power and the implications for world order are considered.

## Introduction

COVID-19 is definitely not the first or the worst epidemic to cause a global crisis, yet it has tremendous implications for International Relations (IR) and world order. Without doubt, it has challenged the established liberal order and has encouraged scholars to ask whether COVID-19 has consolidated realist beliefs regarding IR that they are state-centered, involve power struggles, absolute gains, the Thucydides trap and so on. At the domestic level, there have been calls for a stronger role for central governments. Globally, it has been questioned whether the existing institutions and organizations (e.g. WHO) have failed to deal with the epidemic effectively. Generally speaking, public trust in authority, the media and scientific announcement has been damaged. This is a fragile moment for world order. One journalist, Derek Thompson, even tweeted that “there are no libertarians in a pandemic”. Kissinger also claimed that the coronavirus pandemic will forever alter the world order [1].

It is still too early to decide whether the rising nationalism and isolation caused by the pandemic will scatter global integration and cooperation. However, one thing is certain: this pandemic could be a watershed moment in a changing world order. But *how and in what way?* The article argues that the key lies in China’s role. Against the background of a rapidly rising China, this article assumes that in the post-pandemic era, the role of China in organizing world order will be further manifested. Yet, the world’s liberal order will not be altered radically, and instead, a more authoritarian sub-order will emerge to coexist with the present order. That is to say, it is believed that the emerging world order will be positioned somewhere along the spectrum between constitutionalized and authoritarian orders. However, the world order is not static. Its position in the spectrum depends on critical events and the actions of major states embedded within the international systemic structure.

To support the argument, rather than concentrating on material power, this article looks at the narrative power of China in foreign policy, and its development and application of narrative power in the international sphere. To be more specific, as its main concerns it asks: how has China developed its own narrative?; what narratives have been provided or prompted by China amid the pandemic?; what are the reasons for such narratives?; and what are the implications for world order? Narrative is constructive and has a function in meaning-making and perception-altering, which brings with it great additional effects. A narrative tells a story concerning particular events, it seizes and demonstrates experiences with which an audience can relate and empathize [2]. Narrative construction can very likely “structure and exercise power over the subsequent discussion of issues as well as the policies adopted to deal with them” ([2]: 388). Generally speaking, the role of narratives in International Relations has long been recognized [2, 3], yet the nature of narratives and the processes by which they are constructed by the actors and perceived by the receivers have not been thoroughly explored. The literature, for instance, focuses primarily on the links between domestic narratives and foreign policy, or on Western powers as the major actors [4–6].

Accordingly, in this paper, the author seeks to explore China’s international narratives concerning the COVID-19 pandemic as a diplomatic strategy to alter global perception and their possible influences on the emerging world order. It begins with a brief literature review concerning the changing world order, then moves on to the theoretical content which explores the essence, role and power of narratives. To link theory to evidence, the paper adopts an analytical framework involving *three levels of narrative* (international system, national and issue narratives) and *three aspects of the communication process* (narrative formation, projection/diffusion, and reception)^1^ to study the empirical cases and examine carefully the narrative power exercised by China amid the pandemic crisis. China’s own political strategic background, its embedment within three levels of narratives and its empirical communication process (formation, projection and reception) will be scrutinized. To conclude, the paper will review China’s role and its influence on the formation of the new emerging multi-complex world order, from the perspective of the country’s narrative strength in dealing with the pandemic crisis on the international stage.

In terms of method, this article will rely on a two-level document analysis of state documents, policy speeches, newspapers, magazine articles and (social) media accounts [8]. The first level involves a systematic review of news articles, journals and literature on Chinese narrative power and changing world order. With insights derived from this review, related primary documents such as official statements, policy proposals, speeches, and interviews delivered by government/officials (particularly relevant to the COVID-19 crisis) will be investigated [8]. Besides this, social media (Twitter, YouTube, et cetera) will be used as an auxiliary resource because the pandemic is a current and ongoing issue and the role of social media in narrative discussion should not be ignored.^2^ Then the second level, the discourse analysis, will be integrated with knowledge derived from the first level to establish the “interweaving links between the texts and the...contexts” ([8], [9]:1756).

In brief, the contributions of this paper will be multifaceted. Theoretically, the paper takes an inter-disciplinary approach (International Relations, Communication, and Economics) to consider China’s narrative strength as a means of managing information, attracting attention, and establishing discourse dominance. By utilizing social media, this paper provides a more up-to-date analysis to include the views of the public. Practically speaking, by interweaving China’s narrative in foreign policy and public diplomacy with the context of shaping world order, the article provides an angle from which to ponder potential transformation of the ‘intangible’ power of narratives into virtual influence upon real-world politics – something that is largely underestimated both in academic and practical fields. Stressing the narrative perspective and the coexistence of the liberal and authoritarian orders also adds value to and supplements the existing literature, in which material strength is foregrounded and a dichotomy between liberal and authoritarian order is manifested.

## Literature Review

In the existing literature, there are two main stances concerning the transformation of world order. First is the claim that the existing established liberal order is in temporary crisis and being temporarily challenged, although it maintains that the liberal international order will not easily retreat [10–12]. The idea is backed and can be understood in terms of the ‘path dependency’ effect, in which the choice of menus for further future institutional change or design will be constrained by prior decisions [13]. An international order of institutions and laws is based on “formal rules, compliance procedures and standard operating practices that structure relationships between individual units of the polity and the economy” ([14]:155). Once an international order is established and institutionalized, it is ‘sticky’, that is, it will have lasting influence thereafter and will not easily be altered. While admitting that the existing order is in crisis and being challenged, these authors stress that the role and power of the US need to be further consolidated and strengthened (both domestically and internationally) in order to survive the crisis and to avert major chaotic order. Ikenberry, for instance, argues that no matter how deep the crisis of liberal internationalism runs, the liberal order will not easily be replaced because the operation and organization of world politics is fundamentally grounded on the more general organizing ideas and impulses of liberal internationalism [11]. Challenges may encourage the rethinking and reinvention of liberal order, but not make it vanish. By the same token, for Nye, the concern that the rapid rise of China is a threat to the current liberal order is misguided and overestimated [10]. Liberal order will be sustained because “China is unlikely to surpass the United States in power anytime soon and because it understands and appreciates the order more than is commonly realized” ([10]:13). Admitting that resurrecting the existing order will be impossible and insufficient, Haass believes that “the world is not yet on the edge of a systemic crisis” ([12]:30), and presumes that the role of the US needs to be further strengthened.

Those who adopt the alternative stance concerning the transformation of world order believe that there is a long-term shift in the global system, in which the liberal international order is already irretrievable and suffering from a “grave systemic crisis” [15, 16], and therefore the world order will be more diversified as well as pluralistic, giving way to different mixtures of (sub)orders [17–19]. By claiming that the liberal order has entered a period of accelerated transformation, or perhaps even dissolution, supporters of this stance also try to explore what might take the place of liberal order and shape the new world order. As Kreuder-Sonnen and Zangl put it, “post-Westphalia not only reflects constitutionalist principles of democracy and the rule of law, but also authoritarian principles of autocracy and arbitrary rule” ([17]:3). In stating that “the era of liberal hegemony is past”, Acharya claims that the foundations of the liberal order have been eroded for some time ([18]:283). Yet, instead of being overturned completely, the current order would be partially preserved and merged into the new ‘multiplex world’, “in which elements of the liberal order survive, but are subsumed in a complex of multiple, crosscutting international orders” ([18]:272). While they are open to the potential for different compositions of world order, these authors do not reach consensus about the major actors in the new order. For Kreuder-Sonnen and Zangl, most importantly, the international organizations will become, and evolve to be, independent bodies exercising authority (both in a constitutionalized and authoritarian fashion) [17]. With respect to the weakening ability of all governments, although believing in the dominant liberal order, Nye at the same time stresses that the major challenge to the current order comes from “a general diffusion of power away from governments toward nonstate actors” in the era of the information revolution ([10]:13). For Acharya, the new emerging order will “prepare to live without significant U.S. support” ([18]:280), rely more on the emerging powers (e.g. India and China), and be anchored more by South-South linkages.

The relevant literature has provided thoughtful insights concerning world order. Among others, one of the important concerns is what kinds of new order will emerge. Despite various arguments (US-led, IOs-centered, Multiple-oriented, Rising powers-led, etc.), authoritarianism has received great attention in the literature in contrast with international liberalism [11, 12, 17, 18, 20]. Alongside current world politics and international affairs (Trump’s foreign policy, Brexit [21], the proliferation of populist, nationalist and xenophobic strands in Europe and the US., etc.), the recent COVID-19 pandemic serves as the most obvious example that “varieties of ‘new authoritarianism’^3^ rise to new salience in countries” ([11]:7). As Bond and Gostyńska-Jakubowska observe, most EU governments have restricted fundamental rights in order to tackle COVID-19 [23]. Authoritarianism seems to be the most likely competitor for existing liberalism.

This paper agrees with the viewpoint that the world order is undergoing a transformation which will lead to an emerging order characterized by a plurality of fundamentally contradictory (sub-)orders coexisting in parallel [17]. Yet, instead of stressing that the international order will be organized by internationalized authority structures [17, 24, 25], it argues that sovereign states (state-centric governance) will still be the major components that guide world order; that it will be a structured world order with decentralized authority rather than some form of superior authority. Among others, the role of China will be decisive in determining the direction in which world order will evolve.^4^ Without neglecting the importance of the path-dependency effect of the exiting order, this paper would like to draw more attention to examining *critical junctures,*^5^ like the pandemic, which might play a crucial role in driving the transformation of world order. Last but not least, in addition to identifying with most literature that considers authoritarianism as a potential order that is becoming increasingly prominent, this paper does not aim to consider authoritarianism as an order that will compete to replace the existing one, but as an emerging sub-order in the process of transformation that could have great impact on the making of the new world order. As Acharya puts it, a new emerging order “is not a singular global order, liberal or otherwise, but a complex of crosscutting, if not competing, international orders and globalisms” ([18]:277).

### Constitutionalism and Authoritarianism

Before further discussing the possibility of emerging authoritarian order during the COVID-19 pandemic and identifying China as the major player to push it forward, the paper would like to briefly illustrate the conceptions of two world orders: constitutionalism (liberal order) and authoritarianism.^6^ With regard to the legal procedural question, the distinction between liberal and authoritarian order lies in how political authority should be constituted and constrained [17]. From the perspective of constitutionalism, the authority is constituted in a democratic way and further constrained by the rule of law. By contrast, authoritarianism advocates “the autocratic constitution and arbitrary operation of authority” ([17]:7). To be more specific, for constitutionalism, all acts of political authority should be compatible with individual liberty [17]. “Personal autonomy is prior to any purpose of the state” ([26]:20). Thus, to ensure the proper use of state authority, the promulgation and enforcement of positive law are necessary. It is the democratic legislative and judicial processes that define political authority that construct the special virtue of constitutionalism, which “lies not merely in reducing the power of the state, but in effecting that reduction by the advance imposition of rules” ([26]:23). This constitutionalism is deemed to be the cornerstone of the current liberal order, with its set of institutions, rules and norms, established to ensure and promote peace, prosperity and democracy.

In contrast to constitutionalism, authoritarianism takes *stability* prior to individual liberty.^7^ Most crucially, the underlying principle of realizing stability lies in stable political order to secure public safety. Authoritarianism adheres to the belief that *stability of political order* is the most important element for individual and collective security, particularly in cases of emergency [17]. A breakdown in political authority will be considered as a root of anarchic conditions and a serious threat to peace and security [27]. Thus, authoritarianism supports the centralization and concentration of powers in executive authorities to guard stability of political order for enhancing security and assuring a stable society [17]. That is, the responsibility for stability lies in centralized government authority and the “tradeoff between security and individual liberty should be rebalanced in favor of the former” ([20]:51).

Under authoritarian order, the authorities “ought to do whatever is necessary, not what is legal” to ensure stability ([17]:6). In order to maintain political stability and to realize stability, the authority is exercised in an autocratic and/or arbitrary fashion. To be more specific, there are two fundamental features. Firstly, authority relations are structured vertically with some form of superior authority “whose acts are legally binding also without or even against the constituents”; second, the political authority is not necessarily constituted or constrained by law, which suggests that the power-holders are basically granted with unlimited discretion to accomplish the necessary of maintaining stability, especially in times of emergency ([17]:4). The authorities might, for example, expand executive authority, increase areas of secrecy and state privilege, or expand domestic surveillance [20, 28]. This is the alternative state, according to Kay, that is “premised on some collective purposes” and is “a rationally regulated cooperative engagement,…which refers to the repressive character of a totalitarian regime” ([26]:20). Authoritarianism is pro-authority and community, but against disruption and disorder [16]. In brief, rather than having a democratic constitution and constraint by the rule of law, authoritarian authority is very likely constituted by an act of autocratic self-empowerment and not necessarily exercised in accordance with legal constraints concerning the preservation of stable and peaceful order [17, 27].

Harari reminds us that what will matter most in overcoming this pandemic are the long-term consequences of decisions currently taken by governments and policy makers [29]. The pandemic has forced the world to stand at a crossroads, choosing between “totalitarian surveillance and citizen empowerment” as well as between “nationalist isolation and global solidarity” [29]. Although many fundamental beliefs, ideologies and concepts between constitutionalism and authoritarianism are not compatible with each other, when it comes to a new emerging world order that features a complex system of crosscutting international (sub-)orders coexisting in parallel, a renewed ideological encounter needs to be anticipated: the distinction would not be as simple as democracy good and authoritarianism bad [30]. Rather (and especially at the critical junctures), the importance lies in the soft strength required to promote and narrate ideas that help to stave off uncertainty and avert or survive a crisis. Just as Jentleson states, within societies, across cultures, and among political systems, “a healthy dose of soft power will go to whichever system shows its own people and the world that it can meet these challenges” ([30]:3). The following will describe a theoretical framework for treating narratives as a source of soft power, an approach that could help international actors to tell a good story to increase their influence in the international realm.

## Theoretical Background: Essence of Narratives and Narratives as Power

### Essence of Narratives

#### From Discourse to Narrative: Constructivist Perspective

The notion that data and information are dependent upon narrative interpretation to be meaningful is in accordance with the constructivist epistemology: knowledge is socially constructed [31]. If knowledge is socially constructed, it is also possible to reconstruct and reinterpret [32]. Discourse and narrative are the key to the process of knowledge reconstruction and reinterpretation.

Michel Foucault (1926–1984) first proposed the idea of discourse power as an independent concept [33], and it is used as an approach to understanding discourse as “ways of constituting knowledge, together with the social practices, forms of subjectivity and power relations which inhere in such knowledges and relations between them” ([34]:108). Concentrating upon discourse power aims not only to reveal how concepts became comprehensible, but also to consider what power relations are manifested. As a social construct, discourse is produced and perpetuated by those who possess the power and mediums of communication [35]. In addition, as Diamond and Quinby point out, discourse functions as “a form of power that circulates in the social field and can attach to strategies of domination as well as those of resistance” ([36]:185). As Hutcheon underlines, discourse is not only a tool of domination; rather, it is an instrument of power [37].

In the international sphere, discourse power is mediated through actors like institutions and nations to demonstrate its “regime of truth” [38, 39]. According to Foucault,

Each society has its regime of truth, its ‘general politics’ of truth: that is, the types of discourse which it accepts and makes function as true; the mechanisms and instances which enable one to distinguish true and false statements, the means by which each is sanctioned, the techniques and procedures accorded value in the acquisition of truth; the status of those who are charged with saying what counts as true ([40]:131).

Once a regime of truth is established, the corresponding techniques and procedures will be developed to infuse and ingrain the values that are considered to be true [35]. “Discourses are more than ways of thinking and producing meaning. They constitute the ‘nature’ of the body, unconscious and conscious mind and emotional life of the subjects they seek to govern” ([34]:108). Accordingly, in the context of International Relations, Foucault argues that the external function of discourse is to organize world order [33]. By the same token, Campbell claims that “it is possible to construct, in its own terms, a competing narrative [discourse] that denaturalizes and unsettles the dominant way of constructing the world, thus prying open the space for an alternative interpretation concerned with the entailments of identity” ([41]:172). A nation that is able to control discourse on the world stage will be granted the right to organize world order and to assume power. Reinforcing the view that discourse is power, Foucault [42] reminds us that although discourse is able to produce, transfer and strengthen power, it can at the same time undermine and expose power, making it unstable and possible to thwart [35]. In brief, international society can be seen as an arena for a discourse-power struggle.

Discourse is closely connected with narrative.^8^ In a broader sense, discourse is a(n interactive) part of narrative. Narrative is one expressive means of discourse used to create a shared meaning for one phenomenon among a group of people. Among other things, narrative emphasizes how one actor tells a story that centers on the self. “Narrative is a particular structure made up of actors and events, plot and time, and setting and space” ([7]:256). Discourse is deemed as “the raw material of communication that actors plot into narratives” ([7]:9). Discourses have a structuring influence on narrative action. “Actors reflexively work with discourse to construct narratives with the instrumental aim to influence the opinions and behavior of others” ([7]:10). In brief, narratives refine or reprocess discourses and discourses empower narratives to be influential.

Another important feature of narratives is that they are chronological; they represent events that occur in sequence [43]. They pinpoint “a very specific form of experience based around a sense of time and movement” ([7]:255). According to Abbott, “narrative is the representation of events, consisting of story and narrative discourse; story is an event or sequence of events (the action); and narrative discourse is those events as represented” ([44]:19). This temporal dimension enables narratives to take actors from one status quo to another [7]. That is, in trying to understand an event, one looks at how it started its influences and possible future occurrences.

Moreover, crucially, both narrative and discourse function within a context and they need that context in order to provide a meaningful interpretation of events and stories told. To be more specific, in the sphere of International Relations, both discourse and narrative can be used as tools for nations to tell their stories and experiences, to create an international reality, and to make sense of how the world and international politics operate. Thus, it is important to bear in mind that “narratives, of course, are highly selective and purposefully constructed. Any narrative will omit some parts of the story while emphasizing the other” ([45]:612).

#### Components and Structure of the Narrative

Narratives are constructed, but how? Drawing from Burke’s discussion, this paper outlines the following key components of narratives in order to explain their overall structure and to argue that narratives are power resources [46]. In short, a narrative needs an actor, a setting, an action, and a goal or intention [47].

First, actors and their characteristics. Actors are essential and central to narratives. Being associated with the international system, they can include, amongst others, states, great powers, non-state actors, non-governmental organizations, or multinational corporations. Actors within narratives are allied to characteristics, interests and behaviors [48]. Actors are characterized not only by their own self-presentation but also by how others perceive them. The derived characteristic leads to the creation of an actor’s reputation. Therefore, actors make an effort to frame their own character, and those of others, to promote a specific interpretation of those characters [7]. The nation-state is taken as a major actor in this paper and its analysis of narrative power, but it is important to point out that other actors like state-owned media, diplomats, and national spokesperson, who act on behalf of the state, are substantial channels of a state’s narrative power.^9^

Second, narratives need a setting and environment, otherwise referred to as the stage where actions take place. Different settings can alter the range of issues that need to be resolved as well as the goals that need to be realized. The underlying principles and assertions of the international environment in this paper are those of an interdependent and globalized but fragile and insecure world that has lived through a pandemic, and this puts trust, cooperation and communication between the nations at stake.

Third is conflict or action. How would the actors in such settings react? This highlights not only the importance of temporality (past experience, present situation and future outlook), but also the need to identify perceived threats and to address by whom and how these threats should be countered [48]. The COVID-19 pandemic has its timely features. As they faced first the outbreak, then the spread and finally the easing-off of the pandemic, states have encountered uncertainty and fear of the unknown and have eagerly sought a model or a ‘standardized’ solution. Given that the climate of opinion is ambiguous concerning the crisis, a narrative that is proactively constructed would be particularly influential.

Lastly, narratives contain clear goals and intentions. Narratives are an attractive means of presenting a resolution to realize a goal. They are a form of action that provides possibilities for resolving the disruption to the status quo. In the absence of past experience, for many states, every lesson counts. It is a learning process where what other countries have done, what policies others adopt, and so on, are significant. In this regard, China is an important pioneer or reference point, not only because it had to deal with the corona epidemic/pandemic earlier than others, but also because of its previous experiences with SARS in 2003. As the pandemic has moved on, the narrative concerning the resolutions adopted by China is decisive and has prompted consideration of the possible emerging order that can better cope with the crisis. Simply put, as Miskimmon et al. suggest, narratives entail “an initial situation or order, a problem that disrupts that order, and a resolution that reestablishes order, though that order may be slightly altered from the initial situation” ([7]:7).

In addition to the above mentioned components, according to Miskimmon et al., there are three different levels of narrative that need to be differentiated to understand the overall structure of narratives: International System Narratives, National Narratives and Issue Narratives [7]. International System Narratives elaborate the nature of the structure of international affairs: how the world is structured, who the players are, and how it works. National Narratives focus more on a nation’s story and the values and goals attached to the story. This level of narrative is more concerned with “the identities of actors in international affairs that are in a process of constant negotiation and contestation” ([7]:10). Finally, Issue Narratives “are strategic in the sense of seeking to shape the terrain on which policy discussions take place” ([7]:11). Issue narratives explain why a particular policy is needed and should be adopted as well as how this policy will be successfully carried out [48]. Crucially, the three levels of narrative do not work independently from each other. Instead, they are all connected. Narratives employed at one level may influence narratives at other levels.

#### Ideas as the Substance and Mainstay of Narratives

Having understood the components and structure of narratives, it is vital to explore their inner content: ideas. The relations between ideas and narratives are mutually constructed, reinforcing and closely intertwined.

According to Schmidt, ideas are the substantive content of narratives [49]. Without ideas, narratives would be empty, shallow, meaningless and impotent. Without narratives, ideas will not be dispersed and cannot survive. Ideas are taken as switches for interests, road maps, focal points, strategic constructions, strategic weapons in the battle for control and so on [49–52]. Ideas are used for policymakers to create policies, programs and philosophies (beliefs) [49].^10^ Two types of ideas are contained in the polices, programs and philosophies: cognitive and normative ones. Cognitive ideas explain“what is and what to do,” whereas normative ideas suggest “what is good or bad about what is” in light of “what one ought to do” ([49]:306). Ideas have a crucial role to play in constituting political action and exercising power of persuasion in political debate. Yet, generating ideas is not sufficient, it is necessary to communicate ideas and spread them to the general public for discussion and deliberation. Such communication explains why some ideas successfully become the policies, programs, and philosophies that dominate political reality while others do not. Ideational success and change depend on communication process, in which narratives act not only as a carrier to deliver and present ideas but also as a ‘filter’ to embellish, interpret and promote ideas. Most crucially, during the process, it involves trust-building, and formation of collective action and identity. Narratives are structures of collective that orient individuals’ actions toward the corporate idea [53]. That is, “individuals accept the obligation to act jointly on behalf of collective beliefs, whether or not they subscribe to them personally” ([53]:219). Once a bright idea of portraying the situation is formed and a narrative is constructed with the “discourse begins about what ‘we’ should do, a collective can form much more quickly, leading to ‘swift trust’” ([53]:347). Therefore, narratives act as idea-facilitator to urge people to engage in the “‘ideological labor’- talk, discussion, education, myth-making, and so on- to create a shared representation of the interdependence and the “we” that it constitutes, before anyone has made any behavioral decisions at all” ([53]:346). It is through narratives that ideas turn into “not merely the shared beliefs held by individuals at any given moment (though they depend on those beliefs), but inherently historical phenomena which are kept alive through the generations by an on-going process of socialization and ritual enactment” ([53]:163). The shared beliefs are the aforementioned philosophies, which are the most fundamental ‘deep core’ to undergird the policies and programs [49, 54].

Goldstein and Keohane distinguish three types of belief: world views, principled beliefs, and causal beliefs [52]. In accord with constructivism, they suggest that ideas are as important as interests when explaining human action and affecting policy formation. First, according to Goldstein and Keohane, “world views are entwined with people’s conceptions of their identities, evoking deep emotions and loyalties” ([52]:8). When ideas appear in the form of a world view, they have the greatest impact on human action. For instance, new ideas or conceptions can lead to a new international order, as shown by the Peace of Westphalia in 1648, the emergence of sovereign states led to a new international order dominated by independent nations. Second, a principled belief “consists of normative ideas that specify criteria for distinguishing right from wrong and just from unjust” ([52]:9). The justification of principled beliefs depends largely on larger world views. They act like a bridge to mediate between world views and particular policy conclusions, and transform basic doctrines into instructions for contemporary human action [52]. They have a profound impact on political action. Lastly, causal beliefs are “beliefs about cause-effect relationships which derive authority from the shared consensus of recognized elites” ([52]:10). Causal beliefs offer direction for individuals on how to realize their goals. They are understandable and valued only when they are embedded within the context of broader world views, where principled beliefs are shared.

The three types of belief provide a roadmap which explains how ideas are transformed into narratives that have an impact on the formation of strategies and then become embedded in institutions. First of all ideas spread among individuals, especially political elites, and reach consensus in favor of one particular stance so that the causal belief is established. Then they move to the level of principled beliefs that entail normative bindings, so that more individuals, groups and countries integrate them into their belief system for making judgments and telling right from wrong. Finally, they become world views, establishing and guiding the direction of world order.

### Narratives as Soft Power

The essence of narratives is fundamental. More than that, to comprehend how communication, persuasion and influence in the form of narratives function on the international stage, it is necessary to analyze their role as well as their power in International Relations. To be precise, narratives should be considered as one source of power exercised by international actors, specifically, nation-states.

#### Ideational Resources Matter As Much as Material Ones

Generally speaking, power implies the ability to achieve the outcome one expects by affecting others’ behavior. Barnett and Duvall write that “power is the production, in and through social relations, of effects on actors that shape their capacity to control their fate” ([55]:45). To be more specific, “power means an ability to do things and control others, to get others to do what they otherwise would not” ([56]:154). Such ability to control others is closely related to the possession of certain resources, which can be further converted into political power to influence others’ behavior.

After the Cold War, Nye pointed out that the new international environment would require new ways to influence the emerging new world order [56]. According to Nye, there are essentially three ways to affect others’ behavior in compliance with one’s own wishes and purpose: by threats of coercion (sticks), by inducements (carrots), or by co-opting [57]. The first two behaviors are categorized as hard power and the last as soft power. Soft power has then been put forward as a sophisticated tool or resource for nations to use to develop or maintain their influence.

In contrast to hard power, the concept of soft power describes the capacity to shape or change the preferences of others by attraction rather than force. Namely, by winning hearts and minds rather than wars. Soft power is non-coercive. Soft power engages inherent qualities, such as a non-violent nature or the possession of values, with communication skills (i.e. diplomatic skills) to attract followers. The soft power of a country rests on three intangible assets: its culture, political values and foreign policies [58]. An international actor could be influential by applying its soft power and using the ‘balance of influence’ to shape “strategic circumstances by virtue of status, membership, normative standing, or other persuasive abilities” ([59]:147). Accordingly, the exertion of influence should not be confined to the use of material resources (military and economic ones) to coerce or bribe others. When assessing a state’s influence and power, ideational sources are as important as material ones. Thus, successful states need both hard and soft power – the ability to coerce others as well as the ability to shape their long-term attitudes and preferences [58, 60].

#### Strategic Narrative as a Power Resource – Putting Soft Power into Practice

While it is important to understand that soft power is central to our appreciation of International Relations, it is necessary to go further and to learn how to put soft power into practice. For this we must identify the resources and the operational processes involved in soft power, and recognize the conditions under which soft power resources can be used to define foreign policy and defend national interests.

Similar to the 1990s, due to the information/communication revolution (the emergence of the internet and social media in the twenty-first century) and the power shift (rising China) in international politics, it is time again to ask, what are the best methods of influencing international affairs? In keeping with this new environment, Nye agrees that international affairs in an information age “may ultimately be about whose story wins”([61]:100, [62]:53). “Great powers try to use culture and narrative to create soft power that promotes their advantage” ([3]:20). Yet, as Hayden observes, the ways in which “soft power resources are vested with rhetorical capacity… are not elaborated in most depictions of soft power” ([63]:51). Setting off from this point, the article further adopts the idea of the economics of attention and deals with strategic narrative as one source of soft power, as Roselle et al. suggest [48]. In so doing, it allows a more convincing and tangible grasp of power and influence than an analysis of soft power alone.

As mentioned, narratives are stories that were created to make sense of human lives and environments. They help to shape the way we perceive the social and material worlds, and thus give people an orientation to act upon the surrounding environment. As an overarching term, ‘strategic narratives’ are important political tools with which to “construct a shared meaning of the past, present, and future of international politics to shape the behavior of domestic and international actors” ([7]:2). As Roselle et al. argue, strategic narratives lead us to consider “what means and methods of persuasion and influence are likely to work under what conditions, and to a focus on those conditions of communication and interaction, which have changed so fundamentally” ([48]:71). The concept of strategic narrative is particularly relevant in International Relations at the present time. Especially, in a time of crisis, when nations and political leaders are unprepared or ill-prepared for the chaotic order brought by the complexities and uncertainties of the crisis, narratives play an even more substantial role in managing disorder into order. Strategic narrative is an important part of state power, which is a crucial indicator of a country’s strength, international influence, and appeal. In short, strategic narrative provides a different way of considering and redefining soft power as the capability to: set out the stage for shared understanding; reinforce interactions and then bring about conformity around shared meaning; and/or legitimize one’s actions in prompting or encouraging others to do something [48]. Such soft power is mainly used to reduce tension, alleviate conflict and search for common ground in international affairs. In short, strategic narratives are a communicative tool for achieving political objectives. They integrate interests and goals.

Furthermore, to stress the importance of narratives and perceptions in the information age, especially in the context of international politics, there is a need to take account of the idea of the economics of attention, which treats attention as a capital good and emphasizes the cognitive effects of an information strategy [64]. Given the time-consuming aspect of overflowing information, attention has become a substantial economic resource for perception-building and decision-making [65]. The simple but fundamental connection between information and attention is observed by Simon:

What information consumes is rather obvious: it consumes the attention of its consumers. Hence a wealth of information creates a poverty of attention and a need to allocate that attention efficiently among the overabundance of information sources that might consume it ([66]:40).

Given the limited attentional capability of humans, how to use the mechanisms or means to structure, shape and orient human attention is of prime importance [67]. In brief, information is measured in terms of the attention it earns [64, 65]. Among other things, what is important is the management and utilization of the (dis)information to gain or distract international attention, and then transforming that attention into the cognitive output of other actors so that one nation’s reputation can be maximized. Not only does this focus on promoting positive narratives and suppressing criticism, it can also push disinformation to sow confusion and local discord and to divert blame.

On the basis of the above-mentioned theoretical framework, the following section of the article will investigate China’s narrative power in the midst of the pandemic. With the help of the communication process framework, the formation, projection and reception of China’s pandemic narratives will be studied, with three different levels of narratives considered in the background.

## China’s Narrative Power in the Midst of the Pandemic: China as a Main Actor in the Pandemic Narratives

### Strategic Background and China’s Characteristics: The Political Regime Under Xi’s Administration

China’s leadership under President Xi Jinping is working up efforts to make the Chinese Dream come true and, further, to turn China into a great, responsible power again [68]. China has entered a new era under Xi’s administration, which is characterized by centralized top-down governance and by more outward-looking strategies [69]. In this ‘new era’ under Xi’s administration, state-party integration is the key.^11^ The leadership launched a massive structural reform program to fully integrate state and party organizations. For Xi, the more output-oriented approach, which was adopted by his previous predecessors, did drive economic growth, prompt societal opening and increase local governments’ autonomy; yet, this approach came at the expense of loss of central control and sustained Chinese Communist Party (CCP) leadership [69].

To maintain political stability and the CCP’s capacity to govern China to be rich and powerful, Xi sees the necessity of a unified governance system under tighter party leadership and ideological guidance. As Grünberg and Drinhausen put it, “Xi is returning the party to the fore by rebuilding a centralized, hierarchical system around himself as core leader” ([69]:3). A multi-centered power structure at Politburo level or the so-called intra-party democracy is no longer appreciated and adopted [69, 72]. Under the banner of ‘law-based governance’, CCP’s rule and ideology are codified in laws and regulation, aiming to solidify the party’s hold on power [70]. The expected outcome is an efficient administration steered and supervised by top-level officials to deliver better services and forestall opposition to CCP rule [69].

Such domestic institutional reforms since 2013 under Xi’s administration have great implications at the international level. China has transformed its role on the international stage from embracing pragmatic gradualism to advocating more confident and ambitious strategies. Instead of upholding Deng’s low-profile principle of ‘biding time and hiding strengths’, bolder assertions are favored for the sake of protecting and projecting China’s interests [73]. It is in this context that the pandemic crisis is tackled more seriously and proactively by China through exercising its narrative power internationally. For China, the coronavirus crisis is not only a health emergency, it is also an economic crisis, as well as an international embarrassment. For Chinese leadership under Xi’s administration, the coronavirus is a severe socio-political crisis that could pose a threat to the Communist Party as well as Xi’s popularity and legitimacy. As in the case of the SARS outbreak, China has been criticized internationally for having delayed the identification and announcement of this contagious epidemic.

Many claim that dictatorships are vulnerable to epidemics and other disasters [74–76]. The main reason for this is that the authoritarian regime tends to conceal information and top officials only realize how serious the situation is when it is too late to introduce effective preventative measures, and this can only lead to the exacerbation and spread of disease. In brief, people are not allowed to sound the alarm, or they will be punished for doing so. The system is relatively fragmented and rigid, not only lacking the participation of civil society, but placing too much emphasis on upward accountability and performance-based legitimacy [77]. Government officials are not responsible for the public but for their higher authorities, and local government performance is mainly measured by tangible assessment of economic development and growth.

Certainly, political regime can play an important role in understanding the development of international epidemics. Yet, fairly speaking, declaration of an epidemic is politically and economically sensitive, given the concerns about potential economic repercussions, the risk of social unrest and even the loss of trust in health institutions and central government. Without any active transmission, a government has no incentive to proactively investigate and look for infected cases and then to declare an epidemic. These are particularly important concerns for political regimes like the CCP, whose legitimacy and popularity are based on economic development and people’s trust in ‘almighty’ communist party-governance.

In a similar vein, regional and international dynamics are also substantial considerations in relation to China’s painstaking narratives concerning the epidemic. Given its emerging position as a great power and its long-standing investment in building a positive reputation as a rising responsible power on the international stage,^12^ China would not be willing to endure the embarrassment if international society were to cast doubt on the functioning of the Communist Party. Any arguments or causes that challenge its one-party ruled communist government need to be avoided.

Thus, (re)constructing and reinterpreting the narratives that could alter the perception of China’s international image and the CCP’s legitimacy is as urgent as tackling the pandemic itself.

### Three Different Levels of Narrative: International System, National, and Issue Narratives

Given the previous theoretical background and China’s strategic position, this section investigates China’s narrative strength during the COVID-19 pandemic by looking at how the country has begun to form narratives to exert its influence in response to the crisis. Contextually, this section looks first at the three different levels of narrative which could constrain, regulate or prompt China’s enforcement of its narrative power, and the possible impacts on world order. It then moves on to an empirical examination of the formation, projection and reception of China’s narratives amidst the pandemic.

As mentioned, narratives function with respect to context. China’s narrative power in this paper is considered specifically within the COVID-19 pandemic context. Nevertheless, this situational narrative is embedded within the International System Narrative. As mentioned, the International System Narrative elaborates upon the nature of the structure of international affairs and it describes or defines one state’s place in the international system. Under the current construction of the narrative, the prevalent dominant narrative of world order is defined as constitutionalism and liberalism, which is composed of central characteristics, such as liberal democracy, industrial capitalism, secular nationalism, and open trade, that shape a rule-based international system [78].^13^ Principles such as state-sovereignty and non-interference are established by this liberal order that continue to be central aspects of International Relations [15]. Furthermore, considering the players/actors and how the system works, the leading narratives (concerning power politics, the role of hegemony, a globalized and interdependent world, the importance of cooperation and negotiation in the established international organizations, and the advocacy of peaceful order) imply that when there is a change of power distribution or systematic change, like a power shift and transition, it will inevitably involve violence and conflict, replacement of one order with another, and supersession with new ideas winning over the old ones [79, 80].

In the context of the International System Narrative, a growing China (economically, politically and militarily), as an agent inherent in the system, is narrated with the characteristics of a rising power that might be a challenge or threat to the existing liberal world order and Westphalian system [81]. Most straightforwardly, a narrative concerning the Thucydides trap, a deadly pattern of structural pressure that happens when a rising power challenges a ruling one, is currently prevailing and narrates conflict relations between China and the US, which are destined for war [80]. Against this background, and constrained within the International System Narrative, China is greatly inspired and striving to rewrite and reshape the narrative to improve its national image in the international realm and to prove its benignancy to win greater support for its position.

Accordingly, in its National Narrative, which focuses on its identity in international affairs, China as a rising state has long identified, since its opening up and adoption of an outward facing foreign policy, with the value of peace and has continually emphasized the term ‘peaceful-rising’ to define itself in the international realm. Examples of terminology include, ‘peace and development’ (*heping yu fazhan*), ‘China’s peaceful rise’ (*Zhongguo de heping jueqi*), and ‘peaceful development’ (*heping fazhan*). The National Narrative has evolved from one of a peaceful rising state acting in low-profile to one of emergence as a responsible power that aims to proactively facilitate and promote peaceful order and development.

The concept of the ‘peaceful rise’ was introduced and emphasized at the beginning of the 2000s, under the leadership of Hu Jintao and Wen Jiabao.^14^ The idea was introduced internationally by Zheng Bijian^15^ at the Bo’ao Forum where he talked about China’s strategic path: “This new strategic path is China’s peaceful rise through independently building socialism with Chinese characteristics, while participating in rather than detaching from economic globalization” [83]. Given the constraints of the International System Narrative, and the need to reassure the world that China would rise without being a game-changer and destabilizing the international order, Zheng elaborated explicitly that, “The CCP is not willing to challenge the existing international order, much less has it the desire to break it by using violent forces” [84]. Moreover, the strategic concept of the peaceful rise was explained to have the following five essentials:

1. It would involve taking advantage of world peace to promote China’s development and safeguarding world peace through China’s development;
2. It would be based on China’s own strength and independent hard work;
3. It could not be achieved without continuing the “opening-up policy” and an active set of international trade and economic exchanges;
4. It would take several generations; and
5. It would “not stand in the way of any other country or pose a threat to any other country, or be achieved at the expense of any particular nation” [82, 85].

With the country’s rapid growth and further development in the 2000s, China has realized that there is a growing need for the Chinese government to respond to the ‘China threat’ narrative. Rather than following the previous path of biding its time in obscurity and keeping a low-profile, since 2012, under Xi’s administration, the opportunity for China has come and ‘a rising China’ is now portrayed as more confident, proactive, self-aware, aware of its responsibilities, and less inhibitive of risk-taking [86, 87]. Such a stand was visible at the launch of the diplomatic campaign to promote the narrative advocating the ‘China Dream’, which refers to the great rejuvenation of the Chinese nation, as well as the introduction of the grand project of the Belt and Road Initiative (BRI) to connect Europe with Asia for better global governance [68]. The BRI, presented by China as an international infrastructure development plan, is seen as a communication strategy to formulate the narrative of peaceful development with Chinese influence and aims to change perceptions of the country [88]. To accommodate the narrative of a rising China, the National Narrative has revolved around the idea of ‘peaceful development’ which aims to foreground China’s role as a responsible actor to provide more public good and increase social capital in international society.

President Xi’s statement clearly mentioned that China would “unswervingly take the road of peaceful development and remain a builder of world peace, a contributor to global development and a defender of the international order” [89]. The National Narrative has manifested China’s efforts and its attempts to identify as well as present itself as not just a responsible contributor but also an alternative provider, defending the peaceful and stable world order. Mostly, it implicitly expresses China’s desire to support the plurality of orders. This differentiates China as an alternative provider from a game-changer. China does not aim to lead a revolutionary transformation to alter international rules, principles, norms or code of conduct thoroughly. Nor would China coerce other actors into replacing the international order. Instead, China works on the basis of the existing system to encourage cooperation and global governance, with its expectation of switching world order from a hegemonic to a pluralistic one.

Finally, Issue Narrative is closely related to the COVID-19 pandemic. Working within the constraints of its International System Narrative and striving to define its position and formulate its own narrative in order to win international recognition, China has utilized the issue of the pandemic to develop narratives that not only justify its pandemic policy but also proclaim that Chinese ways and methods are a model of governance worth observing and learning. Policies concerning testing, tracing and lockdown management, travel restriction measures, the mandatory wearing of masks in public, social distancing, border control and blockade, amongst others, are considered necessary and effective. These are short-term constraints for long-term common benefit and welfare. The swift and decisive policy making employed to manage the crisis effectively and establish control over the pandemic is largely attributed to the Chinese style of governance: strong and determined central government that is playing a key intervening role to handle the pandemic properly.

As Zelikow describes, “much of history is punctuated by catalytic episodes. After they happen, people interpret them to construct narratives about past and future” [16]. The COVID-19 pandemic is the main element at play in this catalytic episode and at a historical juncture for China as it attempts to change the global narrative and show the world what works and what does not. As the first country to be hit by the COVID-19 outbreak, China is using its Issue Narrative to share its early experiences as a pioneer and to form a blueprint for other countries. In so doing, China has built its Issue Narrative by giving the world a preview, not just in terms of fighting against the pandemic during the crisis, but also in terms of post-pandemic know-how with regard to corporate responses and strategy following the reopening, operation of the new norm for daily life, and so on. The whole storyline aims to publicize China’s indispensable, beneficial and harmless role in the international realm and to enhance its international image. More detail will be given in the following section which examines the communication process of the Chinese pandemic narratives: their formation, projection and reception.

### Communication Process of the Chinese Pandemic Narrative: Framework of the Empirical Overview, Linking Theory to Evidence

To empirically analyze China’s strategic narrative, this section will review the formation, projection/diffusion and reception of the Chinese pandemic narratives to see how ideas promoted by China are addressed and transformed into the international system. This will help to disclose the soft power China has to reframe the pandemic narrative. Based on the previous discussion, to understand a nation’s strategic narratives, it is a straightforward matter to look at its foreign policy narrative, which can be exemplified through speeches, statements from leaders, and other official claims presented in the media. Additionally, to assess how China’s narrative power is perceived by others and the implications this has for world order, news articles, comments and social media accounts are examined here as a reflection of, and feedback mechanism for, China’s narratives.

#### Formation: China as a Victim, Fighter and Contributor

The formation of strategic narratives is fundamental and substantial as it reveals the strategic goals and types of communication of a given state [7]. The strategic goals can be short-term and/or long-term, and the types of communication include rhetorical argument (persuasion) and rhetorical force (coercion) [7]. China’s broader long-term goal is to cultivate a positive perception of itself over time in the international sphere. To be more specific, it aims to construct narratives that could enhance its appeal to a foreign audience and improve its national image in the international realm during the pandemic. This government-to-public interaction can be associated with public diplomacy that serves to enlarge soft power resources and enhance the attraction of one country [48, 61]. With respect to the communication types, the difference between rhetorical persuasion and coercion lies in power asymmetry and the circumstance of the crisis. While there is a power asymmetry and during the time of crisis, rhetoric is more easily associated with force to “challenge the subjectivity (self) of an actor, thus forcing the target to conform to narratives” ([7]:14). During the COVID-19 pandemic, given China’s rising status and its comprehensive power, the formation of Chinese narratives can be observed more as a rhetorical coercion from one side aiming to acquire compliance via communicative action.

From empirical observation, it can be seen that China has formulated the narratives to shape its positive national image in the international realm. It would be a transformative process to change China’s status/identity amid this pandemic from a ‘*victim/survivor*’ of the pandemic, to a ‘*fighter*’ fighting against the pandemic in a very effective way, and then finally to a ‘*contributor*’ to international society.

China’s victim narrative aims to combat the conspiracy theory spread by Western countries, particularly the US, as well as to refute the accusation of a cover-up. It has tried to deliver the message that China is not the source of the problem and has done nothing inappropriate in terms of dealing with the virus as a member of international society. The virus was not produced in China and it is still not clear where it originated. Officially, a spokesperson for the Chinese Foreign Ministry, Geng Shuang, reiterated that China is a victim of the epidemic and not its accomplice [90]. “It was found in China. It was found in many other places that have no connection with China at all. So you can’t point your fingers at China for the outbreak and we have done our best”; so stated the Chinese ambassador to the United Kingdom, Liu Xiaoming, during an in-depth interview [91]. Responding to suspicions about an information cover-up, Liu further said, “I think China has been straightforward, transparent and swift in terms of sharing information with the WHO” [91]. China‘s ambassador to Canada, Cong Peiwu, also claims that China is being victimized by a campaign of disinformation about its role in the spread of coronavirus [92]. In addition to the official statements, to counter the conspiracy theory spread in Western countries, Foreign Ministry spokesman, Mr. Lijian Zhao, wrote on social media Twitter that “it might be US army who brought the epidemic to Wuhan” [93]. This message was broadly circulated by other official Twitter accounts of Chinese embassies and consulates. Moreover, the state-run China Global Television Network has further intensified and spread the narrative that the virus might have originated from American participants in a military sports competition in October in Wuhan by producing a video in Arabic targeting viewers in the Middle East [94].

While there are narratives in the international sphere stating that China failed to take effective and timely measures to control the transmission of COVID-19 in the early stages so that novel coronavirus spread to other countries, China has realized that the image of a ‘great, responsible, and peaceful power’ that it has tried to establish to push peaceful, prosperous, and harmonious order forward has been damaged. To proclaim that China is an innocent victim, its narrative not only stresses that the enemy is the virus not China, but also attributes the cause to another country. In responding to the international imputation, the victim narrative has evolved from reactive and defensive to active and offensive.

Nevertheless, it is not sufficient for China to be innocent when attempting to further consolidate and restore its positive international image and prove that there was nothing wrong with the actions it took. It is also essential to show the world that the country has tried its best to combat the pandemic. The narrative has developed to stress China’s status as a heroic fighter against the pandemic, alone but persistent and effective. At its core, this narrative praises China’s policy, measures and the actions taken to deal with the pandemic and manage the crisis.

At a regular press conference, Foreign Ministry Spokesperson Geng Shuang firmly made the following comments [90]:

In face of the epidemics [sic], as a signatory to the WHO’s International Health Regulations, China has taken the most comprehensive, strict and thorough measures in an open, transparent and responsible manner, earnestly fulfilled the duties and obligations and adopted and adjusted border exit health and quarantine measures in a scientific and timely fashion in accordance with the Border Health and Quarantine Law of the People’s Republic of China and the Law of the People’s Republic of China on Prevention and Control of Infectious Diseases to prevent cross-border transmission.

To elaborate upon what China had done in an attempt to stem the spread of COVID-19, Mr. Geng presented a detailed timeframe to explain how the lockdown policy for Wuhan city, the health declaration, and quarantine measures all over the country had been adopted in a timely and effective manner. He stated:

Since the outbreak, China has been racing against time and the virus by continuously refining and strengthening prevention and control measures. The Chinese government and people have striven to overcome difficulties and made enormous sacrifices to fulfill due obligations to reduce the cross-border spread of COVID-19 while maintaining anti-epidemic efforts at home. Our important contributions have been well recognized by the international community [90].

By the same token, the Xinhua news,^16^ a representative Chinese news agency that is responsible for “setting the general tone for other media outlets in the coverage of politically sensitive events” ([95]:87), did clearly set the tone:

Quarantining large cities, building a 1000-bed hospital in just 10 days, sharing critical information with the world without delay, and boosting health cooperation with other countries in good faith...In the unexpected battle against the novel coronavirus outbreak, China is making all-out efforts to protect its own people, and meanwhile taking on its responsibility as a major country to contain the spread of the epidemic [96].

In an effort not to allow China’s endeavors to be undermined or underestimated, this fighter-image narrative is being aggressively promoted by Chinese diplomats. According to a report written by Wong and Mozur, in pushing the Chinese narrative, one Chinese diplomat boldly asked the president of the Wisconsin Senate to “pass a resolution recognizing that China has taken heroic steps to fight the virus”, and that resolution has already been proposed with a draft to include lines stating that “China has adopted unprecedented and rigorous measures” and that the actions “have been effective in curbing the virus from spreading to other parts of China and the world” [97]. Similarly, according to the German interior ministry, “The German government is aware of individual contacts made by Chinese diplomats with the aim of effecting positive public statements on the coronavirus management by the People’s Republic of China” [98].

This heroic and fighter image that the narrative tries to deliver is in line with not only China’s strategic aim to shape the perception of the world toward China, but also its deep-rooted face diplomacy, which is a desire for foreign praise and to steer clear of criticism at any cost. Face diplomacy is based largely on Chinese face culture^17^ and the logic of face-nationalism which dictates that its actions are dependent on others voluntarily giving respect, otherwise known as face [99, 100].^18^ China takes the allegations and accusations of other countries as disrespect for the tremendous efforts and sacrifice made by the Chinese people. The country’s self-appointed role has undergone a public challenge amounting to a fundamental loss of face and it has thus responded with more offensive narratives.^19^

China’s efforts to achieve a positive international image and international affirmation as well as recognition have led it to the further construction of its narrative as a contributor amidst the pandemic. As the epicenter of the coronavirus outbreak has gradually shifted from China to Europe and America, China has started to actively construct narratives as a helpful global citizen with new stories about its strength and commitment to the international community [103]. The core value of this contributor narrative is the indispensable role of China as an experienced nation in the international community helping to tackle the crisis. That is, China is a responsible and essential great power. Most importantly, in such narratives, it plays the role of a partner or friend who shares the problems and difficulties with other countries suffering from the pandemic.

The narrative includes the messages that: China has bought time for the world to respond to this health emergency; China has set up the model and is an example for others countries to learn from (for instance, the role of the strong, determined central government, swift and decisive measures such as lockdown policy, building quarantine shelters and hospitals, and mask-wearing obligations); China is willing to offer help to other parts of the world that are suffering (sending delegations of medical and health experts to offer advice); and China acts as a donator (delivering supplies like medical equipment, masks, ventilators), and so on. According to Reuters, Chinese President Xi told United Nations Secretary General Antonio Guterres that “China’s efforts to control the outbreak had given the world *precious time* to formulate their own responses” [104]. According to Xinhua news, China has donated 20 million US dollars to the World Health Organization (WHO), which aims to “support the global fight against COVID-19, and the construction of public health systems in developing countries” [105]. There is plenty of other news available through various channels (video, podcast, newspaper, social media) that publicizes China’s donation of masks and medical equipment to countries like Italy, Iran, Serbia, Cambodia, Ethiopia, and Belgium that have been hit hard by the pandemic.

Besides, on Twitter, Mr. Liu Xiaoming tweeted that “When a vaccine is successfully developed in China, we will make it a global public good. China will join other countries to improve global governance on public health. We will build a global community of health” [106].

The Chinese ambassador to Grenada also wrote a commentary entitled ‘China, an important contributor in fighting against COVID-19’ in praise of China’s approach to overcoming the pandemic:

Since the outbreak of COVID-19 in Wuhan, under the strong leadership of the Communist Party of China and the Chinese government, the Chinese people have united as one, fully brought to bear the strengths of the socialist system with Chinese characteristics, through tenacious efforts and enormous sacrifice, we achieved the initial stage victory against the epidemic. This not only effectively protects the safety and health of the Chinese people, but also greatly boosts the confidence of people around the world in overcoming the epidemic. China has played an indispensable and unparalleled role in safeguarding the lives and health of people around the world [107].

All the contributor narratives highlight China’s goodwill diplomacy and responsible role, and aim to lessen anti-Chinese animosities over the COVID-19 pandemic. In brief, they present the country’s struggles, in an attempt to win international recognition.

From innocent victim, to tough fighter, and ultimately sublime contributor, China has used its soft power to create this storyline, rewrite the narrative of the pandemic and change perceptions about its role in handling the crisis.

#### Projection and Diffusion^20^

It is one thing to conceptualize the strategic goal and to construct narratives accordingly, but narratives in communication are not static but dynamic. It is only through interaction that narratives can be properly delivered and bought into. Thus, formation is just the beginning. How the narrative is further spread and perceived should not be ignored. Interactive and feedback mechanisms decide the effectiveness and sustainability of the message.

To project and diffuse the narratives concerning its image and status amid the pandemic, China is well aware of the significance of preserving their consistency and impartiality and building a shared understanding of their meaning.

First, in order to make its narrative more persuasive and accountable, and to avoid being perceived as arbitrary, China has been circumspect in selecting the ‘speaker’, who further diffuses the narrative. That is, in addition to the statements and official announcements from Chinese diplomats or officials, the country has spared no effort in approaching other foreign channels to voice and transfer the messages from Chinese narratives to make those narratives more objective.^21^ There are two main ways in which China has tried to keep its narratives consistent. One is the mass reports from Chinese media outlets publicizing the compliments and appreciation received from foreign perspectives, which are in agreement with China’s own narrative as a contributor. To list a few examples:

“If it weren’t for China’s efforts, the number of cases outside China would have been very much higher,” said Director-General Tedros Adhanom Ghebreyesus of the World Health Organization (WHO) [108].

The WHO chief stressed that “the speed with which China detected the outbreak, isolated the virus, sequenced the genome and shared it with WHO and the world are very impressive, and beyond words” [108].

“As a result of (China’s) technological development, for example, the sequencing of the virus was carried out in the span of a week. That does not usually happen in these situations,” said Jose David Urbaez Brito, scientific director at the Federal District branch of the Brazilian Society of Infectious Diseases [96, 108].

“China’s rapid sharing of the genetic sequence of the virus is also really important, said Jonathan Gershoni, a virologist and immunologist at Israel’s Tel Aviv University” [108].

The Chinese people “are protecting the world from an even faster spread through their willingness to make sacrifices and their commitment,” said Michael Schumann, head of the German Federal Association for Economic Development and Foreign Trade. “They deserve our respect and our active and energetic support” [108].

“Sherry Rehman, vice president of the Pakistan People’s Party, said Pakistan is learning from China’s experience in taking prompt actions, handling the situation properly and providing services in the face of such an emergency” [108].

The other way is a more advanced but indirect means of supporting the consistency of the Chinese narrative and involves manipulating/managing information [109]. That is to say, in addition to promoting narratives that center on advancing positive and suppressing negative views of China, in order to gain attention and give prominence to China’s rare and commendable role in the pandemic, criticisms have been made and amplified of other actors; criticism of the European Union, for example, for failing to do the same as China has done, in spite of the fact that the EU has actually provided billions of euros and additional crisis response instruments (EU budget flexibility, Emergency support, Safety nets in the EU and EA, etc.) in assistance [109, 110]. Such projection of the narrative has the potential to not only foreground China’s significance but also sow the seeds of discord and division within the European Union [109]. In a similar vein, to magnify China’s contribution and other actors’ failure, an animated video entitled ‘Once Upon a Virus’ is released by Xinhua news agency. This video uses Lego pieces to mock the United States’ coronavirus response and the Trump administration’s claims of an initial Chinese COVID-19 coverup [111]. In brief, through manipulating and managing information, China is able to capitalize on the oversights and mistakes made by other actors to further develop its positive image. All of this is in line with the theory of the economics of attention: the management of (dis)information in order to earn as much attention as possible.^22^

An empirical example can be found in a video recorded in Milan by Eric Sylvers, a reporter from the Wall Street Journal [112]. In his video, there are messages that praise China for supplying assistance and aid amid the COVID-19 pandemic. Italian Foreign Minister Luigi Di Maio publicly expressed his appreciation of the first batch of aid that arrived in Italy from China. Lombardy’s Senior Official, Fabrizio Sala, also made open remarks, saying “Thanks again to China. Thank you (in the Chinese language)” [112]. It is clearly stated in the video that although China’s supplies (it provided facial masks, test-kits, and ventilators, built a temporary hospital in Milan with 400 intensive care units, and dispatched 300 doctors and nurses) might be considered ‘a drop in the bucket’, they were significant and would be remembered by Italians [112]. Italy will remember that China came to its aid when it was in need. For a short time, the hashtag #ForzaCinaeItalia (Let’s Go, China and Italy) was embraced and amplified on Twitter [113]. More important than the praises, in contrast to China’s actions, the European Union seems to have been a disappointment to Italy during the pandemic. The video describes how Italy felt betrayed by the European Union [112].

Sylvers also mentions that there were similar patterns of aid to European countries like Spain, Greece and Serbia to ramp up China’s coronavirus-diplomacy. It is documented in the video that Aleksandar Vucic, Serbian president, made an official public announcement to state that “the Serbian people will never forget this kindness from China”, with Chinese medical personnel standing behind him in the background as a symbolic image [112].

Whether at China’s request or due to the willingness of others to put out such positive messages in praise of China, China’s arduous attempts to use soft power to rewrite the coronavirus narrative and to reshape perceptions about its handling of the pandemic are manifest and discernible.

Second, in terms of projection and diffusion, China’s narrative strength has also encouraged the formation of a *pandemic-crisis identity* in some Asian countries,^23^ which is in contrast to the West. As Subotic explains, “narratives can also be mobilizational, created to establish and promote specific collective values, and encourage a sense of groupness and solidarity” ([45]:612). The pandemic narrative is thus useful in the way that it gives political actors a chance to seize on collectively shared experiences and memories to make specific political points [45, 114]. Collective identity can therefore be formulated on the basis of a group consciousness that shares common goals, actions, attributes and emotional self-recognition [115]. The key point is to highlight commonalities between communities and seek the common ground while preserving the differences at the same time.

In line with China’s victim narrative, when others talk about China’s role in the outbreak, this fuels anti-Asian sentiment to varying degrees around the world (especially in the US where President Trump spoke of the “China Virus” and “Kung Flu”). It has already set the stage for distinguishing Asians from others. Many Asian people caught in the crossfire were then subjected to attack (either verbal or physical). The rhetoric, which was initially anti-Chinese and later spread to become anti-Asian, actually urged Asian people to find common ground, to recognize their common interests and to share their common experiences, which paves the way for the formulation and consolidation of the Asian identity in each community around the world. Thus, the anti-Chinese rhetoric has acted as a prologue for China to seize the opportunity to continue its narrative setting and push the Asian identity^24^ forward during the crisis. This Asian identity is deemed as a common non-Western identity.^25^ It binds the community as a whole “under the banner of a single Asian framework to generate a sense of togetherness, belonging and mutual benefit” [115].

In the midst of the COVID-19 pandemic, another crucial function of government has been brought to light: that of crisis management. In order to respond promptly to rare and overwhelming pandemics, governments need to rely on experts and adopt policies with which their citizens may be uncomfortable. Restrictions and the corresponding enforcements inevitably lead to government’s controversial role in increased surveillance. This is a dilemma that governments have to resolve. As Swee points out, “crisis management requires not only effective policies, but appropriately adaptable governments to deliver them” [118]. It is essential to bear in mind that a central feature of many countries in East Asia since the Second World War has been their ‘developmental state approach’^26^ to domestic and global challenges [119], whereby a top-down management model is adopted by institutions. According to this model, the responsibility for prosperity and stability lies in centralized government authority [120]. States and governments are assigned a relatively strong and centralized role in formulating and pursuing crucial economic and social goals; they possess the strength and ability to mobilize society [121]. Following such processes, and influenced by the model, governments in East Asia (e.g. Singapore, South Korea, and Thailand) have been both better aware of the crisis and relatively flexible and adaptable, enabling them to swiftly enter into crisis management mode to cope with the epidemic.

Faced with the pandemic, many Asian countries have taken similar approaches and measures to combat COVID-19. This is not only because they shared the experience of handling the SARS epidemic, but because they have, to some extent, gone through a similar state-building process based on the so-called developmental state model. Thus, the fighter narrative of China’s initial measures and even its call for cooperation from neighboring countries have not prompted the same level of doubt, criticism or questioning within East Asia as they have in the West (concerning the disregard for civil liberties and human rights). Instead, the fighter narrative has created a sense of crisis management identity in East Asia that foregrounds a stronger role for the state, which is expected to intervene through extensive regulation and planning, and distinguished Asia from the West as more effective and prompt to handle the pandemic.^27^

In the international media, there are many articles, reports and blogs that discuss the Asian experience and the lessons the world can learn from the Asia-Pacific responses to the pandemic [118, 123–125]. Consistent with its narratives, China has also explicitly commented on precious regional efforts to establish regional closeness and common ground through regional narratives:

Chinese President Xi Jinping said that cooperation between China and South Korea against the COVID-19 pandemic is effective and has contributed useful experience to and set a good example of cooperation for the global fight against the coronavirus disease…. The effective cooperation illustrated the principle that ‘a good neighbor is not to be traded for gold’ [126].

According to Xinhua, by the same token, and in response:

Prabowo Subianto, general chairman of the Great Indonesia Movement Party, or Gerindra, and the Indonesian defense minister, said the coronavirus outbreak emergency has prompted huge efforts from governments and medical societies around the world, and the Indonesian people and his party shared the feelings and hearts of the Chinese people and wish them success in their fight against the virus [127].

In addition to shaping the affinity discourse in the region, China has made a great effort to further promote itself as a facilitator and contributor, fostering ‘international cooperation’ to combat the crisis and urging common development and prosperity for a more stable, peaceful and safer world, which is in contrast to the US isolationist and ‘America First’ policies.

President Xi also called for concerted efforts to support the World Health Organization in playing its due role, and cement communication and coordination within such multilateral frameworks as the United Nations, the Group of 20, and the Association of Southeast Asian Nations plus China, Japan and South Korea.

Describing China and South Korea as friendly neighbors that cannot be moved away, he pointed out that the two countries have increasing common interests in promoting common development and prosperity, maintaining regional peace and stability, and safeguarding multilateralism and free trade [126].

Such projection of the narrative seeks to make a conspicuous differentiation between China and the hegemony of the West, in particular the US, advocating China’s way of encouraging cooperation and providing an alternative means of global governance. Accordingly, China can be perceived not only as an alternative provider, but also as a responsible and more reliable partner.

#### Reception

Morgenthau once wrote, “in the struggle for existence and power … what others think about us is as important as what we actually are. The image in the mirror of our fellow’s mind … determines what we are as members of society” ([128]:73). International affairs may possess narrativity, but the story is not complete without the interpretation of the audience. Thus, whether a narrator’s strategic goal can be achieved depends on the reception and perception of their narrative. It is this reception by others that matters when contemplating China’s narrative power in terms of encouraging the emergence of the authoritarian sub-order.

As mentioned in the previous section, China has skillfully invited praise for its achievement from all over the world. Those ‘speakers’ include not only political representatives, but also some virology experts, yet the previous examples show that their affirmative and positive comments are centered on the pandemic and crisis itself. The following are examples of commendations that not only focus on this distinctive single event (China’s pandemic crisis management), but expand to the general performance of the Chinese central government (Communist Party of China, CPC). They imply a certain degree of approval and affirmation of the Chinese political system, which is identified as holding varied beliefs and values toward world order and the international system.

“…after identifying the epicenter of the virus (outbreak), the Chinese government succeeded in isolating millions of people to prevent new infections,” Brazilian infectious disease expert Ana Ferreira said [108].

“This is the reason why the Chinese authorities are forced to quarantine many millions of Chinese people, and I believe this is the only solution”, said Elio Di Rupo, former prime minister of Belgium [108].

“History has repeatedly proven the capability and resolution of the Communist Party of China (CPC) in its leading [*sic*] the Chinese people to overcome various challenges, and he firmly believes the CPC will win again in the fight against the epidemic, said Jurin Laksanawisit, head of the Democrat Party, who is also Thailand’s deputy prime minister and commerce minister. The Democrat Party of Thailand is willing to develop cooperation with the CPC in various aspects, he added” [127].

“Chairman of the Pakistan People’s Party Bilawal Bhutto Zardari said that General Secretary of the CPC Central Committee Xi Jinping and the CPC have led the Chinese people to adopt strong and comprehensive anti-virus measures in a remarkably quick and efficient response to the unexpected virus outbreak, ensuring the safety of both the Chinese people and Pakistani nationals in China. The efforts show the CPC’s firm resolve and outstanding capacity in coping with complex challenges, he added” [127].

“Secretary General of South Africa’s ruling African National Congress (ANC) party, Ace Magashule, said in his message that under the strong leadership of the CPC, the Chinese people are making concerted efforts, with scientists, medical workers, the People’s Liberation Army and other people from all walks of life cooperating effectively” [127].

“First-Vice President of the United Socialist Party of Venezuela Diosdado Cabello said the entire international community has witnessed the decisive actions taken by the CPC and the Chinese government to combat the epidemic. He expressed full confidence that under Xi’s command, the CPC is sure to lead the Chinese people to an ultimate victory” [127].

“General Secretary of the Portuguese Communist Party Jeronimo de Sousa said the CPC and the Chinese government have been actively treating infected patients to prevent the spread of the disease and striving to safeguard the people’s health, and have made immeasurable contributions to containing the spread of the virus around the world” [127].

All these messages are narrative tools which help China to improve its international image and to portray its authoritarian governance system as better equipped than the liberal democratic alternative in the West to handle a crisis.

Yet Chinese narratives are not without their challengers and questioners, and as time goes by, some anti-China narratives have begun to form. For instance, Jude Blanchette, scholar at the Center for Strategic and International Studies, stated that “Beijing may come to regret its rapid pivot from domestic crisis to international triumphalism, for there is already a rising tide of nationalist anger coming its way as citizens from countries around the world face prolonged economic hardship and are in search of culpable parties” [97]. American officials believe that China developed its contributor narrative (shipping medical supplies and donations) specifically to turn the spotlight away from itself as the root of the pandemic. In Europe, there are criticisms and concerns about China’s delivery of shoddy equipment around the world. Political leaders have expressed their skepticism and require detailed, transparent explanations from China concerning the outbreak of the epidemic. One original report, issued by the European Union and documenting how governments push disinformation about the coronavirus pandemic,^28^ stated “China has continued to run a global disinformation campaign to deflect blame for the outbreak of the pandemic and improve its international image … Both overt and covert tactics have been observed” [129]. In the light of China’s image in the international arena, Elizabeth C. Economy, director for Asia studies at the Council on Foreign Relations, pointed out: “what is most striking to me is the extent to which the Chinese government appears to be demanding public displays of gratitude from other countries; this is certainly not in the tradition of the best humanitarian relief efforts” [97]. Regarding China’s claim to make COVID-19 vaccines a public good (particularly for the developing countries), many raise doubts about China’s attempts to use vaccines a diplomatic weapon [130]. This ‘vaccine diplomacy’ will become another means of political pressure to realize China’s national interests. For example, China recently vowed to prioritize the Philippine’s requests for access to successful coronavirus vaccines in return for the Philippine’s moderate stance on the South China Sea issue [131].

Another interesting example of the negative perception of China amid the pandemic is the new online Pan-Asia Alliance formed through the online-transnational narrative #MilkTeaAlliance on Twitter.^29^ This is an online alliance formed by netizens from Thailand, Taiwan, Hong Kong and the Philippines in response to rhetorical attacks from Chinese netizens on Twitter as they push back against perceived critics and defend CCP-endorsed political views (in this case, safeguarding against the coronavirus-related conspiracy theory and protecting China’s stance on territorial sovereignty).^30^ Rather than echoing China’s narrative of Asian identity, these Asian netizens on Twitter came together to show their solidarity in the form of encouraging words and (mostly) memes [132]. In so doing, they found common ground for expressing their anti-China identity. As they wrote on Twitter: “in Milk Tea We Trust” and “Milk Tea is thicker than blood”.

To briefly sum up the empirical communication process, China’s use of narrative power to alter perceptions through an information offensive, to urge the establishment of crisis identity, and to distinguish itself from a hegemony but as an alternative provider, are illustrated from the *formation* and *projection/diffusion* points of view. From the *reception* viewpoint, although there has been some degree of backlash against aggressive Chinese narratives that seek to polish its global image and to speak for its political viewpoints, these criticisms have barely centered on the measures or policies adopted by the Chinese government to handle the crisis. Instead, measures like social distancing, mask-wearing obligations, city lockdowns and so on have been further examined and imitated by many countries in the West (e.g. France, Germany, Spain, Italy, and the US) to fight against coronavirus.^31^ The closer adoption of ‘authoritarian methods’ at the critical moment when managing and governing a pandemic seems to have been tacitly accepted and endorsed.

Most significantly, these negative receptions have not stopped China exercising its narrative power. Instead, the country has become more active (or even taken the offensive) in reshaping its role in the coronavirus story and deflecting blame for the pandemic [113]. China’s narrative power has uncompromisingly undermined confidence in Western governments, which were required to soften and tone down their language when criticizing China’s response to the pandemic. As Apuzzo points out, as long as other international actors, like the European Union or the US, do not have a coherent approach to combating disinformation, China’s narrative power can only be strengthened [129]. Analyzing the communication process (formation, projection and reception) helps to pry into the essence, the motivations, the evolution and implications of China’s narrative strategy in the information age, and shows that China has been building its narrative along with its changing strategic diplomacy – from restrained and low-profile to proactive and assertive.

## Conclusion – Implications for the Emergence of a Post-Pandemic World Order

This article looks at how China has exerted its influence during the pandemic from a soft-power narrative perspective, and considers the possible changes in world order that could occur. It calls for renewed attention to the power of narratives in guiding the social construction of knowledge, yet aims not to ignore the importance of materialist power. As Nye admits, soft power alone cannot guarantee effective foreign policy [133]. Indeed, China’s economic strength has been used as a bargaining chip to bolster its narrative power, as exemplified by a case in which European Union officials softened their criticism of Chinese COVID-19 disinformation and revised a report under heavy economic pressure from China.^32^ With the back-up of hard power, China’s narratives about the pandemic serve as a smart strategy and play a significant role in shaping people’s understanding of the country’s actions in managing the crisis as well as its role in governing world order. They help to define the nature of China’s rise (whether peaceful or threatening).

Due to the proliferation of a variety of new actors and transnational issues the prediction and definition of a new world order is becoming more and more complex. Nevertheless, a successful world order relies on a “balance between legitimacy and power”([134, 135]:9). Power is necessary but it is not sufficient. Legitimacy plays a key role. Legitimacy, however, depends on the prestige of the leading members and some degree of shared values [19]. Narrative is a useful tool for building as well as lifting prestige and promotes the shared understanding of some of the basic social and political values needed to justify legitimacy.

According to Zürn, “both the achievement of common goods (e.g. social welfare) and the way the decision has been made” can serve as sources of legitimacy, which can be used for the construction of legitimation narratives ([136]:70). Amid the pandemic, China’s narrative power gave the appearance that it was working for the common good (reducing the damage done by COVID-19 and providing aid/support in a bid to meet demand), both impartially and internationally, and enabled by its determined system of governance. In so doing, it aims to encourage belief in the legitimacy of its role to react and deal with the pandemic, which further secures its international image and status as a great power in the restructuring of world order. No doubt, this is a rather manipulative process. As Zürn explains, “consumption, glamour, and leader cult also play important roles in such narratives. They usually come with the systematic manipulation of information of what authorities do, and about the circumstances in which they do it” ([136]:76).

On balance, in a world of growing complexity and interconnectedness, the world order is actually an accumulation of ideas and ways of problem-solving [16]. On the battleground of ideas determining the *Tendenzwende* (a German term for ‘change of course’), in order to stand out as victor, a good, useful and trustworthy narrative needs to be conveyed by a persuasive storyteller. Tying in with the pandemic, China has utilized its hard power, the shift in the digital revolution and the rise of a networked world to strengthen its narrative power. Through its own narratives, it has created a general feeling that things are under control in China, but that the rest of the world were on the verge of descending into chaos. This paves the way for China to step in as helper and to plot the scenario whereby it is a required and essential partner and in which authoritarian order is more effective as a means of preserving stability. At this critical juncture, to solve the issues of the pandemic, China has skillfully and softly applied its narrative power to attract attention and to show and remind people all over the world that the old examples and existing order no longer work efficiently to tackle their problems, and another, better, alternative should be offered and seriously considered. China, at this turning point, is not only able but also willing to bear the unshirkable responsibility to ‘correct’ the future world order from a hegemonic to a pluralistic one.

This *alternative* narrative is very strong and convincing. Rather than being a game-changer, China rises with no intention to coerce other actors into replacing the international order; instead, it aims to provide an alternative for other actors to choose freely. This narrative ingeniously allows China to ward off the blame for being the revisionist state and running against the liberal concept of ‘freedom to choose’. Amidst the pandemic crisis, China’s narrative power to question the dominant liberal narratives and to encourage the plurality of world order is meaningful and noteworthy, because what China proposed and publicized could easily turn into a reality once the ‘causal beliefs’ promoted by China’s pandemic narratives have been successfully transformed into ‘world views’ [32, 52, 137]. Carrying the beliefs forward, China’s pandemic narratives “provide the general stimuli and attention-directors that channelize the behaviors of the members, and that provide the members with the intermediate objectives that stimulate action” ([67]:100).

Learning from China’s narratives during the COVID-19 pandemic, it would seem that states are currently the substantial and major actors that govern world order in the international realm and the expectation of a greater dominant role for international organizations is somehow overestimated. The World Health Organization (WHO) is supposed to be at the heart of the battle to tackle and survive the pandemic, acting as a global coordinator shepherding scientific data and experts to fight against COVID-19. However, instead of taking the whole situation into account and planning accordingly, the WHO has been widely criticized for responding slowly^33^ and relying too heavily on China’s one-sided information. Most severely, many experts around the world have raised concerns about the deference of the WHO to the Chinese government and increasing Chinese influence over the institution [138, 139]. The idea that an international organization could be an independent and superior authority with an impartial and constitutionalized essence, able to govern global affairs, is still far from attainable.

To conclude, as Zhao suggests, social practice is essential for one country to gain greater international narrative power [140]. This pandemic crisis has provided a window for China to practice its narrative power and for international society to observe its capabilities. During the COVID-19 pandemic, the development of narrative power to tell a good Chinese story could not only help China to reshape its international image and to strengthen its international legitimacy and status, it could also prompt the emergence of the authoritarian sub-order to coexist in parallel with the dominant constitutional liberal order. At the same time, for China, finding a common shared rule between constitutionalism and authoritarianism is of interest for forging its narrative power ahead. It is therefore crucial to bear in mind that the emerging order should be an inclusive and shared one that aims not to contain or appoint any single power in order to preserve any particular order, but to prevent conflict and the emergence of chaotic order, and to encourage cooperation among countries. Supposing that the world order can be placed somewhere in the spectrum between constitutionalized and authoritarian, this paper argues that China’s narrative power-play during the pandemic might have moved the post-pandemic order closer to the authoritarian end. This position is not permanent, however, but subject to rectification and adjustment by any changes in the international systemic structure as well as behaviors by international actors. In short, the world today is too complex to settle into one single hegemonic order. A world order characterized by a plurality of fundamentally contradictory (sub-)orders coexisting in parallel is to be foreseen. Nonetheless, this does not necessarily imply that a state of chaos or conflict is imminent; rather, this is a pragmatic response to a multiplex world.

## Footnotes

1 The idea is inspired by and borrowed from Miskimmon, O’Loughlin, and Roselle [7].

2 In the empirical part of analysis (either government reports, media outlets, or social media), the paper mainly focuses on the official efforts and channels developed by the Chinese government. The reason is to foreground the role of central state as the major actor to construct and exercise narrative power. Yet, it is noteworthy that the efforts put in Chinese narratives are not limited to the official channels. Many other non-official channels (such as the China-linked Twitter accounts) are also actively involved in the communication process. While they might be more persuasive and effective in diffusing the narratives, these efforts made by non-official channels are largely led or encouraged by central government.

3 The term ‘new authoritarianism’ here refers to the new emergence of various authoritarian features/measures adopted by the democratic regimes, as a backlash against liberalism in countries like Hungary, Poland, the Philippines and Turkey. Among others, several strands like populism, nationalism and xenophobia have gradually proliferated to challenge the liberal democracy [11, 22]. Amid COVID-19 pandemic, quasi-authoritarian measures have been adopted in democratic countries to deal with the crisis. For instance, many EU countries have imposed restrictions on fundamental rights, which cause some public to nurse a grievance against the government and consequently led to the massive protests against coronavirus curb.

4 It is ironic and interesting that while the liberal order seems to be toppling and fraying at the edges, “some of the powers, especially China, that are supposed to challenge it are offering support, albeit qualified and potentially short-term” ([18]:275).

5 Critical junctures are where significant changes happen due to crises, external shocks or other challenges that “call into question existing rules of the game and the repertories of state action” ([13]: 153).

6 The terms as well as concepts are borrowed from Kreuder-Sonnen and Zangl [17].

7 This does not imply that stability does not matter for liberal order. In fact, most orders (including the constitutionalist/democratic ones) count on a certain level of stability. Yet, the paper tries to highlight the extent authoritarianism stresses on stability and security; as well as the way the authority is exercised to realize stability.

8 There are, however, discrepancies between the definitions when it comes to methodology of analysis. Generally speaking, narrative is by definition broader than discourse. Yet, instead of clearly differentiating between discourse and narrative, the article tends to treat both terms interchangeable. Nevertheless, it is vital to point out that given the international structure, strategic concerns of states in IR, and the chronological feature of COVID-19 pandemic, ‘narrative’ is taken as the major subject of this paper and will be used throughout the paper. However, discourse is a part of the narrative that should not be overlooked due to its ‘powerful influence’. Discourses play a crucial role to strengthen and reinforce narratives. In short, the term ‘narrative power’, used in this paper, refers to China’s ability to utilize discourse and narrative to achieve its objectives. China’s *pandemic discourse* needs to be put into a larger ‘narrative’ context to be assessed and understood.

9 While treating nation-state as a major actor, this article does not aim to overlook the role played by private sectors and individuals. It is without doubt that private sectors or individuals are also the substantial actors to exercise narrative power, especially when it comes to narrative diffusion. In the case of China, some private sectors or individuals (although being subject to central government’s narratives) might be considered more persuasive and effective to diffuse the narratives than central government. Yet, from the holistic perspective, the paper takes official efforts (namely, the actions from nation-state) as the major sources to formulate, construct, diffuse, project and reflect the narratives.

10 Policies refer to specific policy ideas or solutions proposed by policymakers; programs are used to underpin the policy ideas; and philosophies are the most fundamental one acting as deep core to undergird the policies and programs with organizing ideas, values, and principles of knowledge and society [49].

11 For more information concerning China’s state-party system, please refer to Jiang [70] and Backer [71].

12 While trying hard to develop its positive image and reputation on the international stage, China is still very sensitive about being ‘humiliated’ or accused by other countries. China’s actions are dependent on others voluntarily giving respect. (This is the so-called face diplomacy. For more details, please refer to the discussion in the section ‘Formation: China as a Victim, Fighter and Contributor’) Once China feels that its efforts have been disrespected, it will become more assertive. Additionally, no matter how important its international reputation is, China’s core interests cannot be threatened or compromised. These might explain China’s growing assertive stands against its neighbors, Australia, the EU and the US. with regard to many different aspects (e.g. COVID-19 pandemic, the Hong Kong National Security Law, and the activities in the South China Sea) in pandemic times.

13 According to Flockhart et.al. the international liberal order was the creation of the United States and Britain and later also continental Western Europe following the end of WWII [78]. The founding of the World Bank, International Monetary Fund, and United Nations in 1944 and 1945 signified the beginning of the order and it has further deepened with the creation of NATO and the European Union. “The idea of liberal international order rests on four key elements: free trade; post-war multilateral institutions; the growth of democracy; and liberal values” ([18]:272).

14 Although the use of this term was queried within the CCP on the grounds that it could be interpreted as too ambitious and give the wrong impression internationally, ‘peaceful rise’ still gained traction as a description of China’s strategic direction of development. There was, however, a suggestion that the country should revert to the concept of ‘peace and development’, which used to be a key principle of its foreign policy dating back to Deng Xiaoping [82].

15 Zheng is a formidable intellectual figure within the CCP [82]. He addressed and introduced the concept of ‘China’s peaceful rise’ in his speech entitled ‘A New Path for China’s Peaceful Rise and the Future of Asia’ at the Bo’ao Forum for Asia in 2003.

16 According to Hartig, Xinhua news is the largest news agency and is commonly known as “the mouthpiece of the CCP and plays a crucial influential role in the hierarchical Chinese media system” ([95]:87). It is an important tool for China to use to exercise its narrative strength.

17 “Face is the figurative self-shown to others” ([100]:67). In the context of Chinese culture, face culture is called ‘mianzi’ culture, which is like a code of honor, and it can be analyzed to reveal how face is gained or lost [100].

18 For more various ideas to understand nationalism, self-image, and China, please refer to Gu [101] and Mikecz [102].

19 China’s recent assertive positions against the US., EU, Australia etc. can be understood from such perspective.

20 As mentioned previously in the Footnote 9, although it might be useful to observe narrative diffusion from the perspective of Chinese private sectors and individuals, the paper limits the observation from official efforts considering the consistency of taking nation-state (Chinese government and officials etc.) as the major actor and its role played in the holistic process of communication (formation, projection/diffusion, and reception). This does not imply to overlook the role of Chinese private sectors and individuals in distributing narratives.

21 As mentioned previously, Chinese diplomats actively approached foreign government officials to make official statements and praise China in public. While the cases of Germany and Wisconsin Senate failed, China still managed to gather individual appreciations from all over the world.

22 Another example of China’s management of disinformation is the campaign in mid-March that it contained texts and social media messages amplified by China-linked accounts to incite public panic and increase distrust in the US. government [109]. The texts and messages spread false warnings about a nationwide lockdown and troop deployments in the US.

23 Broadly speaking, Asian countries here may refer to countries locating in the Asia-Pacific. To be more specific, this paper mainly refers to East Asian countries, which include Northeast and Southeast Asia.

24 Given China’s interest in global governance, pushing the formation of the Asian identity is a crucial step for China to firstly unite the region and ensure a rather stable political climate. Ever since 1990s, China has been increasingly involved in the process of East Asian regionalism and jointly worked with ASEAN, Japan, and South Korea on formulating and strengthening regional identity via regional cooperation. Based on a shared culture and value, the Asian identity helps to distinguish ‘we/us’ from ‘they/others’.

25 The Asian values play a key role to form the Asian identity. The original concept of Asian values was launched by Lee Kuan Yew, the former Singapore Prime Minister, and henceforth invited the fierce debate on the definitions and politics of ‘Asian values’ [116]. Generally speaking, being influenced mainly by the Confucianism, Asian values are featured with preference for collective welfare, social harmony, socio-economic prosperity, a sense of duty, loyalty and respect towards figures of authority and other principles of Confucian moral philosophy [117]. Accordingly, when it comes to the level of international politics, national unity, security and stability are of the utmost importance.

26 It is important to clarify that political ideology or the political system is not what defines the developmental state. Rather, the developmental state approach stresses the relatively strong role of the state and its specific structure, as well as measures taken to lead the process of development, so as to ensure legitimacy, authority and control.

27 With regard to general safety and long-term stability, the Deep Knowledge Group conducted research to rank countries which give their citizens the lowest likelihoods of being infected, the lowest chance of COVID-19 mortality, and the highest likelihoods of recovery and positive health outcomes [122]. From the ranking, based on four major parameters (quarantine efficiency, government management efficiency, monitoring and detection, and emergency treatment readiness), only one European country (Germany) is listed in the top 10 ‘COVID-19 Safety Ranking Framework’, while others are mainly located in the Asia-Pacific region (South Korea, Australia, China, New Zealand, Taiwan, Singapore, Japan, Hong Kong, in descending order).

28 According to Apuzzo, this original report was then revised to remove the sentence concerning China’s “global disinformation” campaign due to pressure from China [129].

29 The name of the alliance is derived from the well-known milk-tea cultures in those places.

30 For more details concerning how a Thai-Chinese meme war led to a new (online) Pan-Asia Alliance, please refer to McDevitt [132].

31 Certainly, the scale and restrictions adopted in China and in the West cannot be placed on a par.

32 For more details, please refer to Apuzzo [129]. The revised report from the EU shows how Beijing reacted swiftly and effectively to tamp down Western criticism of its pandemic response. The EU’s stance is largely dependent on its hopes to win trade concessions from Beijing and restore a rich relationship in the post-pandemic era.

33 For instance, the outbreak of the Corona epidemic was traced back to December 2019 in China, but it was only in March 2020, after the virus had spread to more than 100 countries, that the WHO finally declared it a pandemic.
