# Correction to: the Post-Pandemic World: between Constitutionalized and Authoritarian Orders – China’s Narrative-Power Play in the Pandemic Era

**DOI:** 10.1007/s11366-020-09709-0

**Published:** 2020-12-01

**Authors:** Yung-Yung Chang

**Affiliations:** International Consortium for Research in the Humanities, Friedrich-Alexander-Universität Erlangen-Nürnberg (FAU), Hartmannstr. 14, 91052 Erlangen, Germany

**Correction to: Journal of Chinese Political Science.**

10.1007/s11366-020-09695-3

The original version of the article missed to add two references concerning method added to the main text and in the reference list. Following are the changes on page 3 of the original version as well as the details of the two missing references. The subsequent reference citations were also adjusted accordingly in the original version.

In terms of method, this article will rely on a two-level document analysis of state documents, policy speeches, newspapers, magazine articles and (social) media accounts [8]. The first level involves a systematic review of news articles, journals and literature on Chinese narrative power and changing world order. With insights derived from this review, related primary documents such as official statements, speeches, policy proposals, and interviews delivered by government/officials/media outlets (particularly relevant to the COVID-19 crisis) will be investigated [8]. Besides this, social media (Twitter, YouTube, et cetera) will be used as an auxiliary resource because the pandemic is a current and ongoing issue and the role of social media in narrative discussion should not be ignored.^1^ Then the second level, the discourse analysis, will be integrated with knowledge derived from the first level to establish the “interweaving links between the texts and the…contexts” ([8, 9]:1756).

The original article has been corrected.

## Footnotes

1 In the empirical part of analysis (either government reports, media outlets, or social media), the paper mainly focuses on the official efforts and channels developed by the Chinese government. The reason is to foreground the role of central state as the major actor to construct and exercise narrative power. Yet, it is noteworthy that the efforts put in Chinese narratives are not limited to the official channels. Many other non-official channels (such as the China-linked Twitter accounts) are also actively involved in the communication process. While they might be more persuasive and effective in diffusing the narratives, these efforts made by non-official channels are largely led or encouraged by central government.
